# Assessment of the Electromagnetic Radiation Exposure at EV Charging Facilities

**DOI:** 10.3390/s23010162

**Published:** 2022-12-23

**Authors:** Hongguk Bae, Sangwook Park

**Affiliations:** Department of Electronic Engineering, Daegu University, Gyeongsan 38453, Republic of Korea

**Keywords:** electric vehicle, EV charging, electromagnetic exposure, electromagnetic field exposure

## Abstract

As the number of electric vehicles (EV) increases, the number of EV chargers also increases. Charging infrastructure will be built into our close environment. Because of this, the assessment of the electromagnetic field exposure generated from the charger is an important issue. This paper valuates the electromagnetic field exposure of six EV chargers. To assess the level of exposure of EV chargers, the electromagnetic fields from six chargers were measured and analyzed. In addition, measured electromagnetic field exposure levels were evaluated against ICNIRP guidelines. Higher electromagnetic fields were measured with standard chargers than with fast chargers. For the fast charger in the charging state, the magnetic field increased with the charging current. Electromagnetic field exposures for all six chargers did not exceed standard limits. The results of the assessment of the electromagnetic field exposure of the six EV chargers will contribute to the establishment of standards for the evaluation of the electromagnetic field exposure of the EV chargers in the future.

## 1. Introduction

As the cumulative supply of electric vehicles (EVs) worldwide increased significantly, the charging infrastructure is also developing. According to the *EV Charging Infrastructure Supply Status and Technology Trend Report*, the cumulative supply of EVs in 2018 exceeded 5.1 million units. The relatively low EV sales are expected to increase rapidly and occupy a significant proportion and the electrification of automobiles is a continuous process. Consequently, the global charging infrastructure has increased, with approximately 5.2 million chargers in 2018 [1]. With domestic and international trends, EVs and charging stations are rapidly increasing and are also expected in our surroundings. As regards location, EV charging stations can be installed in any environment with electricity. This includes private homes, apartments, and public spaces. Therefore, it can be predicted that more charging infrastructure will be built in our environment [2,3,4,5].

This has raised concerns about the effects of electromagnetic fields generated from EV chargers on the human body. The interest and concern of the public regarding the severe potential health hazards of electromagnetic fields and other environmental problems resulting from electromagnetic fields such as malfunctioning of other devices are emerging in society. The international special committee on radio interference and the International Organization for Standardization are currently addressing electromagnetic compatibility (EMC) issues in the automotive environment [6]. EMC experts have raised the issue of the adverse effects of electromagnetic fields on the human body in an automotive environment. This issue is also closely attended to by automobile companies, and research and countermeasures on the detrimental effects of electromagnetic fields in the electric-power-based automobile environment should be prioritized. In addition, various problems of harm to the human body may result from electromagnetic fields, such as trade barriers or lawsuits with consumers owing to inter-country regulations in the future. For this reason, studies on electromagnetic shielding are being conducted to reduce or block the electromagnetic field [7,8]. Studies on the EMC of EV charging using wireless power transfer have been studied in various environments [9,10,11,12], however, only a few research studies about the plug-in charging method exist [13,14]. Therefore, in a situation where charging facilities of various specifications are closely located around humans, it is necessary to evaluate the level of exposure of humans to electromagnetic fields from plug-in EV charging facilities to protect public health from damages caused by electromagnetic fields.

This study evaluates the exposure of EV charging facilities to electromagnetic fields. Six EV wired chargers and measurement locations were selected for evaluation. Electromagnetic field measurement results for representative measurement locations for each charger were inspected and the changes in the electromagnetic field measurement value as the changes in the state of charge (SoC) were observed. The relative value was presented and analyzed by comparing the electromagnetic field measurement value with the domestic electromagnetic field human protection standard.

## 2. Materials and Methods

This section introduces the international electromagnetic field protection standards and exposure index and the criteria to evaluate the exposure amount of electromagnetic fields generated from EV chargers. Subsequently, six types of wired chargers are selected, and the measurement method and equipment are described.

### 2.1. Safety Guidelines

The analysis of the exposure to electromagnetic fields in EV charging facilities requires evidence. Most countries regulate electromagnetic field exposure limits based on the electromagnetic field protection standards of international organizations [6]. International standards include the International Commission on Non-Ionizing Radiation Protection (ICNIRP) and the Institute of Electrical and Electronics Engineers (IEEE). In 2002, IEEE revised the safety standards for human exposure to electromagnetic fields in the frequency range of 0 to 3 kHz [15]. In 2005, it was revised for the frequency range of 3 kHz to 300 GHz [16]. Subsequently, in 2014, the guidelines for the protection of military workplaces and military personnel were updated. In addition, IEEE Std C95.1-2019 was revised in 2019 [17,18]. ICNIRP guidelines provide a reference level for electromagnetic field exposure situations. These guidelines were revised for the frequency band up to 300 MHz in 1998 [19], and in 2010, the reference levels for 1 Hz to 100 kHz were revised [20]. In 2020, the time to average measured values in the 100 kHz to 300 GHz band was divided into 30 min intervals, 6 min intervals, and 0–6 min intervals, and the reference level standards were revised [21]. As a domestic standard, an electromagnetic field protection standard was announced by the Ministry of Science and ICT. Table 1 lists the standards for electromagnetic field intensity for the public (related to Article 3, Paragraph 1), and it follows the ICNIRP guidelines revised in 1998 [22]. Therefore, in this study, the electric and magnetic field strengths were analyzed based on these standards. Table 1 lists the frequency ranges of electric and magnetic field strength baselines. If the level of electromagnetic field exposure exceeds the standard value, it may be interpreted that the human body protection standard is not met. Therefore, in this study, the exposure index was calculated using the reference value. This indicator can confirm the level of electromagnetic field radiation compared to the reference value. The exposure index is described in Section 3.

### 2.2. Measured Charger

Domestic EV chargers can be broadly divided into fast and standard chargers. This is elaborated in Table 2 by dividing them into installation type, charging method, rated output, and charging standard. The standard charger can be divided into a stand-type installed on the floor, a wall-mounted type attached to the wall, and a mobile type that charges by plugging into a 220 V outlet. In the case of fast chargers, they are classified into the charging ports. The charging ports of domestic vehicles can be divided into an integrated type comprising a fast and a standard charging port and a separate type with a different location. The integrated type can either be the direct current (DC) combo type or the three-phase alternating current (AC) type, whereas the CHAdeMO type is employed as a separate type. They are all in accordance with the IEC-62196-2 standard [23]. Other chargers have a non-charging port that is independent of the charging standard indicated in Table 2 (charging port). In this study, six chargers were selected as measurement targets. These are described separately by their rated capacities and installation type. The standard chargers with a rated capacity of 7 kW are stand-type A, stand-type B, and wall-mounted chargers. The 3 kW rated capacity is the standard mobile. In the DC combo method, there is a fast stand-type C with a rated capacity of 50 kW and a short stand-type D with a rated power of 120 kW. In addition, among the six types of EV chargers, standard stand-type A, standard wall-mounted, standard mobile, and fast stand-type C measured the electromagnetic field while charging EV A with a battery capacity of 64 kW. In the standard stand-type B and the fast stand-type D, the electromagnetic field was measured while charging EV B with a battery capacity of 75 kW.

### 2.3. Measuring Equipment

The NARDA EHP-50C was used for electromagnetic field measurement. This equipment displays electric and magnetic field measurements as a frequency spectrum and measures in the frequency range of 5 Hz to 100 kHz. In addition, the reference level of the ICNIRP guidelines corresponding to each frequency band is indicated by a solid red line in the graph. This is further described along with the measurement results in Section 3.2. Two types of measurement display values can be selected in this equipment: actual and RMS mode. This equipment samples every 1 µs. The measurement interval is 250 ms. In the actual mode, the instantaneous value is displayed during the measurement, and it is measured once every 30 or 60 s. In the RMS mode, the calculated root mean square value is displayed by measuring the electromagnetic field strength for the measurement time(s) specified by the user. In this study, the measurement time was set to 60 s. All electromagnetic fields are measured by the Fast Fourier Transformation (FFT) method using this equipment.

### 2.4. Measurement Methods and Procedures

**Figure 1:**
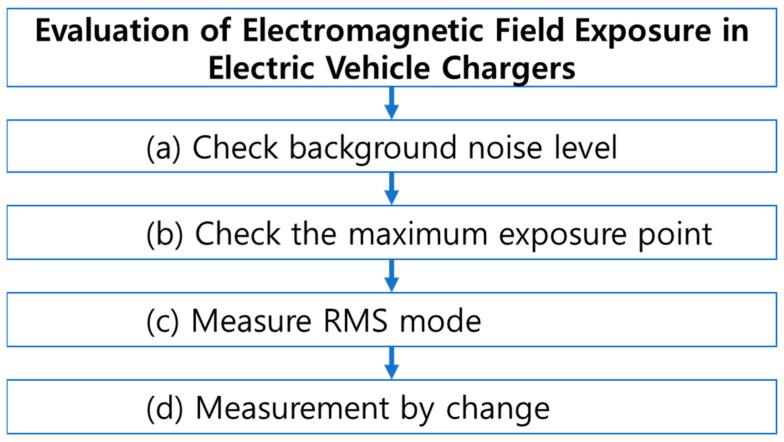
Measurement procedure for evaluation of exposure to electromagnetic fields in electric vehicle charging facilities.

The electromagnetic field exposure from EV charging facilities is evaluated as shown in Figure 1. The measurement sequence and conditions were designated in the order of (a) to (d) and Table 3, respectively. First, the background noise level was checked. This means the measured electromagnetic field value is in an uncharged state. This measurement step is aimed at obtaining accuracy and reliability when measuring in a state of charge. The body of the EV charger, cable, and handle were measured. Second, the location of maximum exposure to electromagnetic fields in the charging situation was determined. This is a step to determine the trend for precise measurement at the location of maximum exposure to the electromagnetic field. Therefore, the entire body of the charger, cable, and handle are measured in the actual mode. Third, measurements are made in the RMS mode for maximum exposure points of the identified electric and magnetic fields. The second and third steps limit the range to measurements ranging from 20% to 80% of the SoC. This is because of the onboard battery charger, designed to prevent EV battery overload. It switches to the constant voltage mode when the SoC exceeds 80% during charging. Because the charging voltage is constant and the charging current gradually decreases, irregular values may be measured when measuring the electromagnetic field. Therefore, it was precisely measured in RMS mode between 20% and 80% of SoC. Finally, the change in exposure level was confirmed as the SoC changed from 0% to 100% during charging. In the RMS precision measurement step, a single position where high electric and magnetic fields are measured was selected. This is the case with a body, cable, and handle.

**Figure 2:**
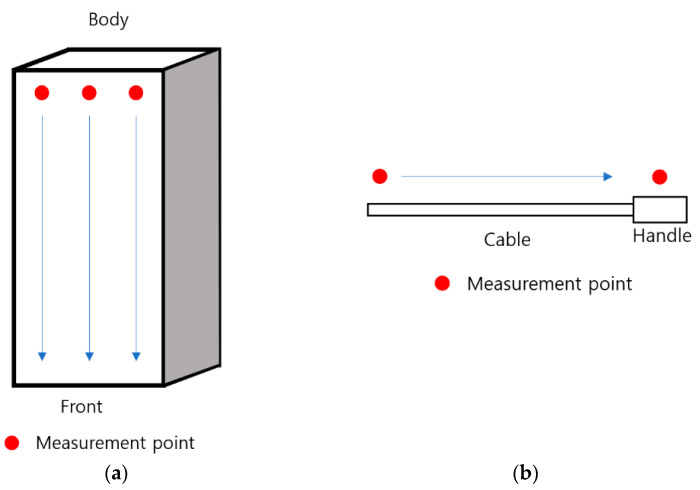
Electric vehicle charger measurement location: (**a**) body; (**b**) cable and handle.

**Figure 3:**
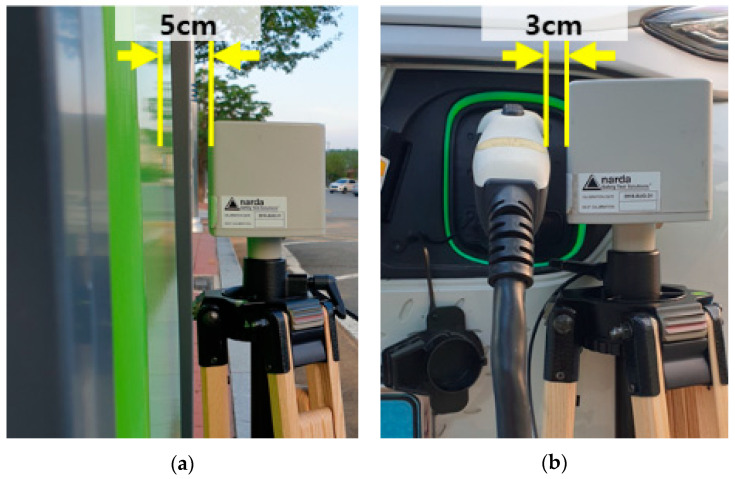
Distance between the meter and charger: (**a**) main body; (**b**) handle.

As shown in Figure 2, the measurement location was divided into the main body, cable, and handle. When measuring the charge, the main body’s front, back, and side surfaces were measured, and the front and back sides were measured by dividing the horizontal axis into three equal parts and moving the probe from top to bottom. As shown in Figure 3, the distance between the charger and the probe was measured at 5 cm for the body and 3 cm for the cable and handle. Other cables and handles were measured at a distance of approximately 3 cm.

## 3. Measurement Results

In this section, the measurement results for each measurement procedure are checked and analyzed. The electromagnetic fields in the uncharged state of six chargers were measured to determine the background noise level. Next, in the charging situation, the entire charger was precisely measured in RMS mode. Thus, the location where the maximum electromagnetic field was measured was selected.

### 3.1. Background Noise Level

Before analyzing the electromagnetic field exposure in a charging situation, the background noise level that may influence the readings is checked. The electromagnetic field generated from the main body, cable, and handle during non-charging was measured. The electric field was measured from a minimum of 0.07 V/m to a maximum of 11.5 V/m and the magnetic field was measured from 0.002 A/m to a maximum of 0.003 A/m. Moreover, the highest electromagnetic field was measured at 60 Hz. The electric field reference value for 60 Hz was 4166.67 V/m and the magnetic field reference value was 66.66 A/m. Compared to the reference value, the electromagnetic field exposure during non-charging is negligible.

### 3.2. Comparison of Measurement Results by Each Charger

**Figure 4:**
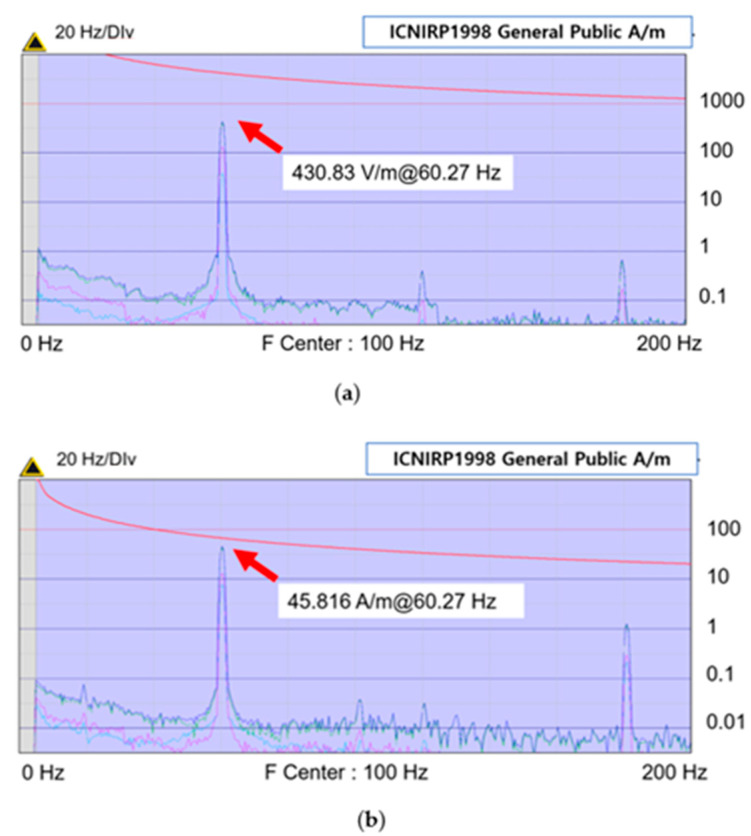
RMS mode electromagnetic field maximum: (**a**) electric field measurement and ICNIRP 1998 General Public reference level; (**b**) magnetic field measurement and ICNIRP 1998 General Public reference level.

Figure 4 is the data graph value saved when the electromagnetic field is measured with NARDA EHP-50C. It is possible to find the value of the highest peak and acquisition method of data. The red line means the reference level value based on ICNIRP 1998 General Public, and visually checking is possible to ascertain whether the measured value exceeds the reference level. The case where the electric field measured in the RMS mode in the charging state is the highest is shown in (a), and the case where the magnetic field is the highest is shown in (b).

Section 3.3 compares the measurement results for each charger. The main body, cable, and handle of the six aforementioned classified chargers classified were measured in actual mode. Among them, the maximum value was precisely measured between 20% and 80% of the SoC in RMS mode and is summarized in Table 4. In the case of a fast charger, the maximum electric field was distributed between 0.21 V/m and 0.65 V/m, and the maximum magnetic field was distributed between 0.062 A/m and 2.58 A/m. In the case of a standard charger, the maximum electric field was distributed between 139.05 V/m and 213.91 V/m, and the maximum magnetic field was distributed between 1.54 A/m and 12.06 A/m.

**Figure 5:**
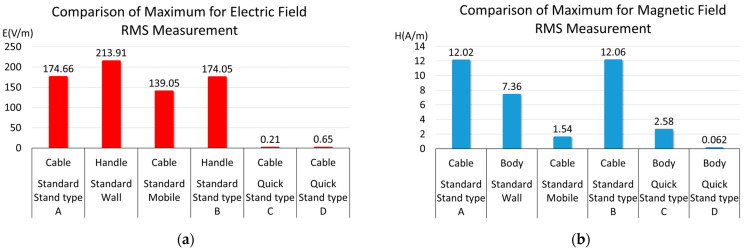
Comparison of maximum RMS measurement value: (**a**) electric field RMS measurement; (**b**) magnetic field RMS measurement.

The graphs of the maximum values of electric and magnetic field RMS measurements for the six types of chargers are shown in Figure 5a,b, respectively. The maximum electric field was mostly measured in cables and handles. The maximum magnetic field was primarily measured in the body and cable. In addition, the electromagnetic field measurement of the fast charger was significantly lower than that of the standard charger.

### 3.3. Comparison of the Maximum Electromagnetic Rankings of the Six Chargers

As mentioned in Section 2.1, the exposure index (EI) was calculated to compare the electromagnetic field measurements with the reference values. The EI was calculated as in Equation (1).

(1) $$\mathrm{EI}\left(\%\right)=\frac{Meas}{Ref}\times 100$$

The EI is the value obtained by dividing the electric or magnetic field measurement value “*Meas*” by the electric or magnetic field reference value “*Ref*,” multiplied by 100. According to Table 1, the reference value needs to be separately calculated based on the frequency range. In this study, the worst-case electromagnetic field exposure situation is considered. The EI is calculated in the frequency band, where the highest electromagnetic field is measured. In addition to the measurement results in Figure 4, the electric and magnetic field maxima at 60 Hz were predominantly measured in all the measurement results. Therefore, the EI was calculated for a single frequency of 60 Hz. The electric and magnetic field reference values for 60 Hz are 4166.67 and 66.66 A/m, respectively.

Table 5 and Table 6 list the rankings based on the maximum electromagnetic field and EI of the six chargers. This includes (c) RMS precision measurement and (d) measurement results by the change in the SoC during the measurement procedure in Figure 1. The electric and magnetic fields of the standard stand-type B and the magnetic field of the standard wall mount exceeded the EI by 10%. Between 1% and 10% are the standard mobile’s electric field, the fast stand-type C’s magnetic field, the standard wall mount’s electric field, and the standard mobile’s magnetic field. Below 1% are the electric and magnetic fields of fast stand-type D and the electric fields of fast stand-type C. The wall-mounted charger with an EI of 69% generated the maximum magnetic field. In addition, the standard stand B charger had the most prominent electric field, with an EI of 10%.

### 3.4. Comparison of Measurement Results by the Change in the SoC

**Figure 6:**
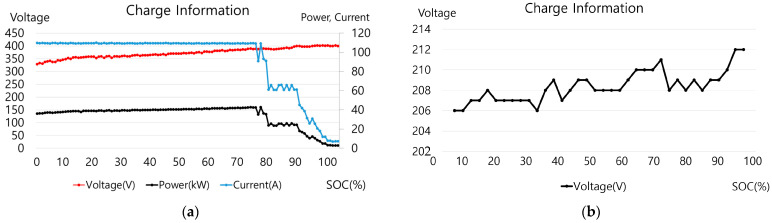
Stand-type charging information: (**a**) fast stand-type C (power, current, and voltage); (**b**) standard stand B charging information (voltage).

In the measurement at the change in the SoC, each case of a fast charger and a standard charger was compared. In the case of high electric and magnetic fields in RMS precision measurement, the main body of the fast stand-type C and the standard stand-type B cable were compared. The charging information by the change in the SoC of the two chargers is shown in Figure 6a,b. For fast stand-type C, the SoC (%), voltage, current, and power from the charger can be checked. In the case of the standard stand-type B, the power, current, and voltage can be checked in the vehicle; the power and current are displayed constantly and only the change in voltage is observed.

**Figure 7:**
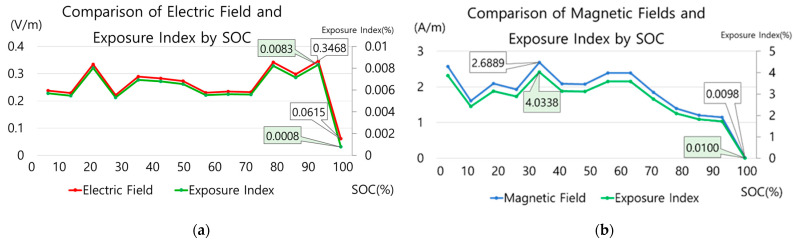
Electromagnetic field characteristics about fast stand-type C main body SoC change (**a**) Comparison of electric field measurement and exposure index (**b**) Magnetic field measurement and exposure index comparison.

**Figure 8:**
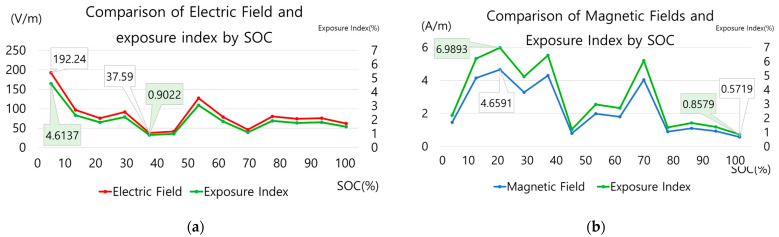
Electromagnetic field characteristics of standard stand B cable SoC change: (**a**) comparison of electric field measurement and exposure index; (**b**) magnetic field measurement and exposure index comparison.

This charging information was compared with the electromagnetic field measurement results to analyze the relationship with changes in the electromagnetic field. Figure 7 is a graph showing the electric and magnetic field measurement values and EI in the main body of fast stand-type C. Figure 8 is a graph of the electric and magnetic field measurements and exposure index in the standard stand B cable. The graphs in Figure 6a and Figure 7 and Figure 6b and Figure 8 can be compared.

In the case of fast stand-type C, electric and magnetic field changes were observed with changes in the SoC. In addition, the correlation between the current and the magnetic field can be confirmed. In Figure 7a, the magnetic field decreased when the SoC was 74%, and the current decreased when the SoC was 77%. That is, the trends of the current and magnetic field graphs are similar. Comparing the EI of the electric and magnetic fields, the maximum EI of the magnetic field is approximately 4% higher.

When the charging information by the SoC and the curves of the electric and magnetic field were compared in the standard stand-type B cable, changes in the electric and magnetic fields were observed with the change in the SoC, where the charging voltage exhibited some change; however, the correlation between the electric and the magnetic field could not be confirmed. Moreover, when comparing the EI of the electric and the magnetic fields, the EI of the magnetic field was approximately 3–6% higher than that of the electric field.

As mentioned earlier, the changes in electric and magnetic fields by changes in the SoC were observed. In the case of a fast charger, the correlation between the change in the current and magnetic field by changes in the SoC was confirmed. In the case of the standard charger, changes in the electric and magnetic fields by the SoC were observed. Therefore, the measurement results of the changes in the SoC when preparing the electromagnetic field measurement for EV wired chargers and the evaluation method of electromagnetic field exposure in the future should be observed.

## 4. Conclusions

In this study, electromagnetic field exposure assessment results of six EV chargers were presented. These results show the location where the electromagnetic field is the highest measured among electric vehicle chargers in the charging situation. They also show the correlation between changes in the electromagnetic field and changes in the SoC. The measured value of the electromagnetic field was analyzed by comparing it with domestic and global electromagnetic field intensity standard. The domestic electromagnetic field intensity standards were in agreement with the ICNIRP guidelines revised in 1998 and were analyzed based on them.

Six EV chargers were selected for measurement. The level of exposure to electromagnetic fields was confirmed by precisely measuring six types of chargers in RMS mode under charging conditions. In addition, the electromagnetic field changed as the SoC changed. Because of RMS precision measurement, a relatively higher electromagnetic field was emitted from a standard charger than from a fast charger. The maximum electric field was measured at the standard stand-type B handle, and it was 430 V/m, corresponding to an EI of 10%. The maximum magnetic field was measured on a standard wall-mounted body and was 46 A/m, corresponding to an EI of 69%. None of the six chargers exceeded the electromagnetic field protection standard.

Changes in electric and magnetic fields were confirmed by the changes in the SoC of six chargers. In the case of fast charging facilities, as the charging power and current gradually decreased, it was confirmed that the level of magnetic field strength decreased accordingly. In the case of standard charging facilities, the correlation between charging power, voltage, current, and electric and magnetic field strength could not be confirmed. In addition, changes in the electromagnetic field were observed with changes in the SoC. Generally, the maximum value of the electromagnetic field was measured in the measurement results by the change in the SoC. This result indicates the need to verify electromagnetic field measurements as the change in the SoC.

Because of these results, the measurement procedure when preparing a method to evaluate the amount of exposure to electromagnetic fields in EV charging facilities is worth reviewing in the future. When charging an EV, the measurement location of maximum electromagnetic field exposure is necessary. Therefore, first, an understanding of the overall electromagnetic field strength of the charging facility is necessary. The maximum value after measurements according to the change from 0% to 100% of the SoC at the location of maximum exposure is worth recording.

## Figures and Tables

**Table 1:** ICNIRP guidelines’ reference levels for general public exposure (1998).

Frequency Range	$E-\mathrm{Field}\;\mathrm{Strength}\;(V\;m^{-1})$	$H-\mathrm{Field}\;(A\;m^{-1})$
up to 1 Hz	-	3.2 × 104
1–8 Hz	10,000	3.2 × 104/f2
8–25 Hz	10,000	4000/f
0.025–0.8 kHz	250/f	4/f
0.8–3 kHz	250/f	5
3–150 kHz	87	5
0.15–1 MHz	87	0.73/f
1–10 MHz	87/f1/2	0.73/f
10–400 MHz	28	0.073
400–2000 MHz	1.375f1/2	0.0037f1/2
2–300 GHz	61	0.16

**Table 2:** Classification of domestic electric vehicle chargers.

Charger Type		Charging Method	Rated Power	Charging Port
Fast Charger	Standard	DC	DC 50 kW/500 V/100 A	CHAdeMO
			DC 50 kW/450 V/110 A	DC combo
		AC	AC 43 kW/380 V/63 A	AC 3-phase 7-pin
		DC	DC 120 kW	Non-charging port
Standard Charger	Standard	AC	AC 7 kW/220 V/32 A	AC single-phase 5-pin
			AC 7 kW/220 V/80 A	Non-charging port
	Wall Mount		AC 7 kW/220 V/32 A	AC single-phase 5-pin
	Mobile		AC 3 kW/220 V/12 A	AC 3-phase 7-pin

**Table 3:** Conditions for the measurement procedure.

Measurement Procedure	SoC (%)	Measurement Position	Measurement Mode
(a)	Uncharged	Body, cable, and handle	RMS
(b)	20~80	Body, cable, and handle	Actual
(c)	20~80	Body, cable, and handle	RMS
(d)	0~100	One of body, cable, or handle	RMS

**Table 4:** Comparison of maximum RMS measurement values of 6 types of wired chargers.

Charging Location		Charger	Standard Charger Type A	Standard Wall Mount	Standard Mobile (Body-Control Box)	Standard Stand- Type B	Fast Stan— Type C	Fast Stand- Type B
	E/H							
Main	E(V/m)		40.15	178.61	28.54	111.98	0.1	0.26
	H(A/m)		0.28	7.36	0.55	1.71	2.58	0.06
Cable	E(V/m)		174.66	183.93	139.05	144.40	0.21	0.65
	H(A/m)		12.02	1.51	1.54	12.06	1.41	0.06
Handle	E(V/m)		132.59	213.91	114.13	174.05	0.04	0.37
	H(A/m)		4.94	0.87	0.08	4.20	0.1	0.54

**Table 5:** Summary of the maximum ranking of the electric field measurement results.

Max Rank	E (V/m)	Exposure Index (%)	Charger	Measurement Position
1	430	10	Standard stand-type B	Handle
2	389	9.3	Standard mobile	Cable
3	241	5.8	Standard wall mount	Handle
4	0.71	0.017	Fast stand-type D	Cable
5	0.40	0.010	Fast stand-type C	Handle

**Table 6:** Summary of the maximum ranking of magnetic field measurement results.

Max Rank	H (V/m)	Exposure Index (%)	Charger	Measurement Position
1	46	69	Standard wall mount	Body
2	12	19	Standard stand-type B	Handle
3	5.2	7.7	Fast stand-type C	Body
4	2.3	3.4	Standard mobile	Cable
5	0.13	0.2	Rapid stand-type D	Cable

## Data Availability

Not applicable.
